# Unsupervised Integration of Multiple Protein Disorder Predictors: The Method and Evaluation on CASP7, CASP8 and CASP9 Data

**DOI:** 10.1186/1477-5956-9-S1-S12

**Published:** 2011-10-14

**Authors:** Ping Zhang, Zoran Obradovic

**Affiliations:** 1 Center for Data Analytics and Biomedical Informatics, Temple University, Philadelphia, PA 19122, USA

## Abstract

**Background:**

Studies of intrinsically disordered proteins that lack a stable tertiary structure but still have important biological functions critically rely on computational methods that predict this property based on sequence information. Although a number of fairly successful models for prediction of protein disorder have been developed over the last decade, the quality of their predictions is limited by available cases of confirmed disorders.

**Results:**

To more reliably estimate protein disorder from protein sequences, an iterative algorithm is proposed that integrates predictions of multiple disorder models without relying on any protein sequences with confirmed disorder annotation. The iterative method alternately provides the maximum a posterior (MAP) estimation of disorder prediction and the maximum-likelihood (ML) estimation of quality of multiple disorder predictors. Experiments on data used at CASP7, CASP8, and CASP9 have shown the effectiveness of the proposed algorithm.

**Conclusions:**

The proposed algorithm can potentially be used to predict protein disorder and provide helpful suggestions on choosing suitable disorder predictors for unknown protein sequences.

## Background

Identification of regions in proteins that do not have unique structures, called intrinsic disorders, is addressed computationally by a number of groups that aim to predict this property from sequence information [1-10]. Contrary to the lock and key paradigm, disordered regions were recently found to be involved in many important functions [11] and in various diseases [12].

Computational characterization of disorder in proteins is appealing due to the difficulties and high cost involved in experimental characterization of disorders. The first predictor of protein disorder was developed by our group in the year 1997 [13]. Due to the importance of predicting this property, in the year 2002, protein disorder prediction was introduced as a category of the CASP contests [14], which promoted the development of new methods for prediction of protein disorder. Consequently, the number of prediction methods available through the Internet has increased rapidly. More than 50 predictors of intrinsic protein disorder have been described in a recent review by He et al. [15], enabling researchers to use a meta approach to predict protein disorder by integrating the prediction results of several methods. Recently, four such meta predictors, i.e. metaPrDOS [16], MD [17], PONDR-FIT [18], and MFDp [19], have been developed for the purpose of improving disorder prediction accuracy. They showed significantly improved performance in performed experiments as compared to using individual component predictors.

A limitation of these supervised learning based meta predictors is that they are prone to over-optimization in their integration processes since they are developed relying on disorder/order labeled training datasets that contain a very small number of proteins that have not already been used for development of the component predictors (e.g. sets as small as the DisProt [20] or as specialized as missing coordinates from the PDB [21]). Therefore, the prediction results of previous meta predictors may not be so good for proteins that have sequence patterns very different from cases used for integration. For example, although it achieved higher prediction accuracy than all predictors participating in CASP7 as stated in its paper [16], metaPrDOS failed to be one of the top predictors in CASP8 [22]. Moreover, one of metaPrDOS' component predictors, i.e. DISOPRED [2], was more accurate than metaPrDOS in CASP8 [22].

To address potential over-optimization problems of meta predictor development by learning from small labeled data, here we introduce a new disorder meta prediction method. By following the idea from Raykar et al. [23] we derived an iterative MAP and ML estimation (MAP-ML) based algorithm for the construction of a meta predictor in a completely unsupervised process using protein sequences without confirmed disorder/order annotations. Performance evaluation of the new meta method is presented by using CASP prediction targets as the test sets, which enabled us to compare the prediction results with other methods used in the CASP contests.

## Methods

### Problem and statement

Let us define the dataset as . Here, ***x****_i_* is an amino acid composition feature vector which is derived from the subsequence covered by a moving window centered at the i-th amino acid within the current protein.  (1 represents a disordered state while 0 represents an ordered state) is the prediction label assigned to the instance ***x****_i_* by the j-th predictor. M is the number of predictors. N is the number of amino acids in the protein.

The first task of our interest is to estimate the sensitivity (i.e., true positive rate) ***α*** = [*α*^1^,…,*α^M^*] and the specificity (i.e., true negative rate) ***β*** = [*β*^1^,…,*β^M^*] of the M predictors. The second task is to get an estimation of the unknown true labels *y*_1_,…,*y_N_*.

### The proposed MAP-ML algorithm

To fulfill the two tasks defined before, we propose an iterative algorithm that we will call MAP-ML. Given dataset *D*, we use majority voting to initialize the probabilistic labels *μ_i_* (i.e., the probability when the hidden true label is 1). Then, the algorithm alternately carries out the ML estimation and the MAP estimation which are described in details in the following subsections. Given the current estimates of probabilistic labels, the ML estimation measures predictors’ performance (i.e., their sensitivity ***α*** and specificity ***β***) and learns a classifier with parameter ***w***. Given the estimated sensitivity ***α***, specificity ***β***, and the prior probability which is provided by the learned classifier, the MAP estimation gets the updated probabilistic labels *μ_i_* based on the Bayesian rule. After the two estimations converge, we get the algorithm outputs which include both the probabilistic labels *μ_i_* and the model parameters *θ* = {***w***,***α***,***β***}*.*

The proposed iterative MAP-ML algorithm is summarized in Algorithm 1, and the estimations are described in the following subsections.

Algorithm 1 (Iterative MAP-ML Algorithm)

**Input:** Protein sequences with prediction labels from M predictors.

**Output:** The estimated sensitivity and specificity of each predictor; the weight parameter of a classifier; the probabilistic labels *μ_i_*; the estimation of the hidden true labels *y_i_*.

**Step 1** Convert the protein sequences into amino acid composition feature vectors.

**Step 2** Use majority voting to initialize .

**Step 3** Iterative optimization.

(a) ML estimation – Estimate the model parameters *θ* = {***w***,***α***,***β***} based on current probabilistic labels *µ_i_* using (1) and (3).

(b) MAP estimation – Given the model parameters *θ*, update *μ_i_* using (8).

**Step 4** If *θ* and *µ_i_* do not change between two successive iterations or the maximum number of iterations is reached, go to the Step 5; otherwise, go back to the Step 3.

**Step 5** Estimate the hidden true label *y_i_* by applying a threshold on *µ_i_*, that is, *y_i_*=1 if *µ_i_* >*γ* and *y_i_*=0 otherwise. Here use *γ* =0.5 as the threshold.

### ML estimation of the model parameters

Given the dataset *D* and the current estimates of *µ_i_*, the algorithm estimates the model parameters *θ* = {***w***,***α***,***β***} by maximizing the conditional likelihood. According to the definitions of sensitivity and specificity, we get(1)

Given probabilistic labels *μ_i_*, we can learn any classifier using ML estimation. However, for convenience, we will explain it with a logistic regression classifier. By using that classifier, the probability for the positive class is modeled as a sigmoid acting on the linear discriminating function, that is,(2)

where the logistic sigmoid function is defined as *σ*(*z*) = 1/(1 + *e*^–^*^z^*). To estimate the classifier’s parameter ***w***, we use a gradient descent method, that is, the Newton-Raphson method [24](3)

where g is the gradient vector, H is the Hessian matrix, and *η* is the step length. The gradient vector is given by , and the Hessian matrix is given by .

### MAP estimation of the unknown true labels

Given the dataset *D* and the model parameters *θ* = {***w***,***α***,***β***}, we define probabilistic labels . Using the Bayesian rule we have(4)

which is a MAP estimation problem.

Conditioning on the true label *y_i_* ∈ {1,0}, the denominator of formula (4) is decomposed as(5)

Given the true label *y_i_*, we assume that  are independent, that is, the predictors label the instances independently. Hence,(6)

Similarly, we have(7)

From (2), (4), (5), (6), and (7), the posterior probability *μ_i_* which is a soft probabilistic estimate of the hidden true label is computed as(8)

where

### Analysis of the MAP estimation

To explain how the MAP estimation model works, we apply the logit function to the posterior probability *µ_i_*. From (8), the logit of *µ_i_* is written as(9)

where  is a constant. The first term of (9) ***w****^T^****x****_i_* is a linear combination (provided by the learned classifier) of the current amino acid’s composition features. The second term of (9) is a weighted linear combination of the prediction labels from all the predictors. The weight of each predictor is the sum of the logit of the estimated sensitivity and specificity. From (9), we can infer that the estimates of the hidden true labels (in logit form) depend both on protein sequence information and on the prediction labels from all the predictors.

## Results

### Evaluation criteria

CASP evaluation was based on per-residue predictions of the entire set of targets. The performance of predictors was evaluated by three criteria: the average of sensitivity and specificity (ACC), a weighted score (S_w_) that considers the rates of ordered and disordered residues in the datasets, and the area under the ROC curve (AUC).

In CASP, predictors were asked to submit a binary label of “O” or “D” (order or disorder state) and a probability that the specific position is in a disordered region (a value in the range of 0 to 1) for each residue. The binary classification of each predictor was assessed by the following scores:

where TP is the number of true positives (disordered residue that were classified correctly), FP false positives (ordered residues that were classified as disordered), TN true negatives (ordered residues that were classified correctly), and FN false negative (disordered residues that were classified as ordered), respectively. The higher the two scores, the better the predictions; therefore, they were combined into a single score, which is the average of the two:

Since the disordered residues are rare in the targets, the weighted score S_w_ was introduced at CASP6 [25]:

where the W_disorder_ was the total percent of order and W_order_ was the total percent of disorder. Therefore, S_w_ ranges from -1 to 1 and predicting all the residues in the targets to be ordered would result in a zero. As defined, this measure greatly rewards disordered residues correctly identified as disordered while heavily penalizing any disordered residue that is misclassified.

The ROC curve was used to examine the ability of the predictors to estimate the confidence level of their predictions. The ROC curve is based on the disorder probability parameter. Once the probability is given, by setting different threshold values of the disordered status, the values of sensitivity and specificity will change accordingly. By taking (1-specitificity) as the x-axis, and sensitivity as the y-axis, all the data pairs corresponding to the minimal threshold value to the maximal threshold value will make a continuous curve. This is the ROC curve, the area under this curve (AUC) is a reliable indication for the quality of the prediction. The value of AUC is between 0 and 1, the larger the area, the better the predictor.

### Performance evaluation using the CASP data

To assess prediction performance, we used CASP9 data consisting of 117 experimentally characterized protein sequences with 23656 ordered and 2427 disordered residues. To reduce noise due to experimental uncertainty, in the evaluation process we didn't consider disorder segments shorter than four residues. We have also obtained prediction labels with disorder probabilities of all predictors which participated in CASP9 from the contest's official website [14]. We selected 15 predictors developed by groups at different institutions assuming that their errors are independent. We set the size of the moving window as 21 which is based on our previous study [26] as well as the ratio of long (>30 residues) disordered segments to short ones in the data.

In the experiment, as the input of our iterative MAP-ML algorithm we used the sequences of 117 protein targets and the prediction labels from the 15 component predictors. After the algorithm had converged, we used the estimation of the hidden true labels *y_i_* produced by MAP-ML as the binary disorder/order predictions and the probabilistic labels *µ_i_* from MAP-ML outputs as the disorder probability. We also used the majority voting method to integrate the component predictors, so that we can compare that method with the MAP-ML algorithm method to see which one is more effective. The majority voting method assumes all predictors are equally good.

**Figure 1:**
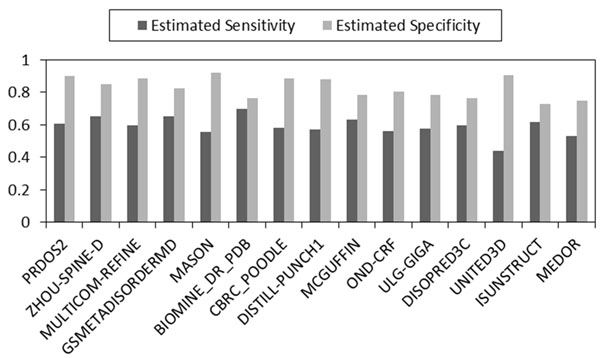
**CASP9 accuracy estimates without using labeled data.** Estimated sensitivity and specificity of 15 disorder predictors is obtained by the MAP-ML algorithm at CASP9 protein sequences without using CASP9 experimentally determined disorder/order labels. The predictors are sorted in descending order of the average of the estimated sensitivity and specificity.

Estimated sensitivity *α* and specificity *β* of 15 component predictors using our MAP-ML meta predictor without relying on true disorder/order labels are shown in Figure 1. The obtained estimates are sorted according to the average of their estimated sensitivity and specificity and were quite consistent with evaluations reported by the CASP9 committee [27] who used labeled data of confirmed disorder/order residues for their evaluations.

**Figure 2:**
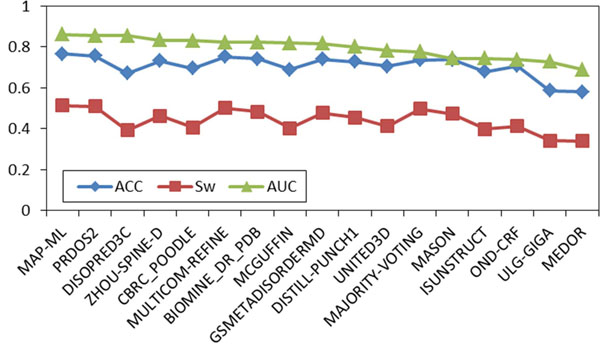
**CASP9 comparison on labeled data.** Evaluation scores are shown for the MAP-ML algorithm, majority voting method, and the 15 component predictors at disorder/order labeled CASP9 protein sequences and the corresponding experimentally determined disorder/order labels. ACC, S_w_, and AUC scores are sorted in descending order of the AUC score.

A comparison of 15 predictors, the majority voting method, and our MAP-ML meta predictor on CASP9 labeled data with confirmed disorder/order is shown in Figure 2. The details of evaluation scores are summarized in Table 1. On this comparison our iterative MAP-ML algorithm had an ACC score of 0.764, a S_w_ score of 0.513, and an AUC score of 0.859. These scores were superior to the 15 component predictors in the CASP9 contest and also superior to the majority voting integration. In addition, Figures 1 and 2 could be used to assess similarity of accuracies and rankings of 15 predictors obtained by MAP-ML algorithm without any labeled data versus their evaluation on true labels by CASP9 committee.

**Table 1:** CASP9 evaluation scores on labeled data.

Predictor Name	Institution^*^	ACC	S_w_	AUC
MAP-ML		0.764	0.513	0.859
PRDOS2	Tokyo Tech, Japan	0.754	0.509	0.855
MULTICOM-REFINE	University of Missouri, USA	0.750	0.500	0.822
BIOMINE DR PDB	University of Alberta, Canada	0.741	0.483	0.821
GSMETADISORDERMD	IIMCB in Warsaw, Poland	0.738	0.476	0.816
MASON	George Mason University, USA	0.736	0.473	0.743
MAJORITY-VOTING		0.735	0.496	0.776
ZHOU-SPINE-D	IU School of Medicine, USA	0.731	0.462	0.832
DISTILL-PUNCH1	UCD Dublin, Ireland	0.726	0.453	0.800
OND-CRF	Umea University, Sweden	0.706	0.412	0.737
UNITED3D	Kitasato University, Japan	0.704	0.412	0.781
CBRC_POODLE	CBRC, Japan	0.694	0.405	0.830
MCGUFFIN	University of Reading, UK	0.688	0.402	0.817
ISUNSTRUCT	IPR RAS, Russia	0.679	0.396	0.742
DISOPRED3C	University College London, UK	0.670	0.391	0.853
ULG-GIGA	University of Liege, France	0.585	0.341	0.726
MEDOR	Aix-Marseille University, France	0.579	0.338	0.688

^*^Only the first author’s institution is shown here as well as in Tables 2 and 3.

**Figure 3:**
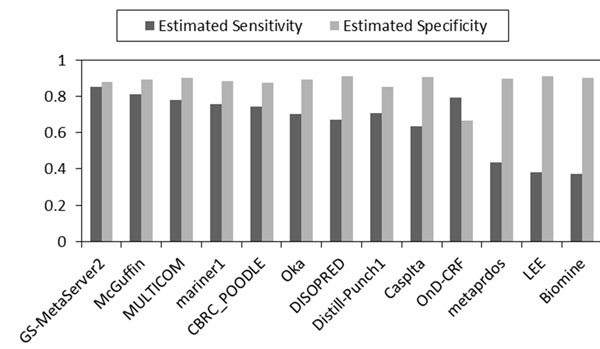
**CASP8 accuracy estimates without using labeled data.** Estimated sensitivity and specificity of 13 disorder predictors is obtained by the MAP-ML algorithm at CASP8 protein sequences without using CASP8 experimentally determined disorder/order labels. The predictors are sorted in descending order of the average of the estimated sensitivity and specificity.

**Figure 4:**
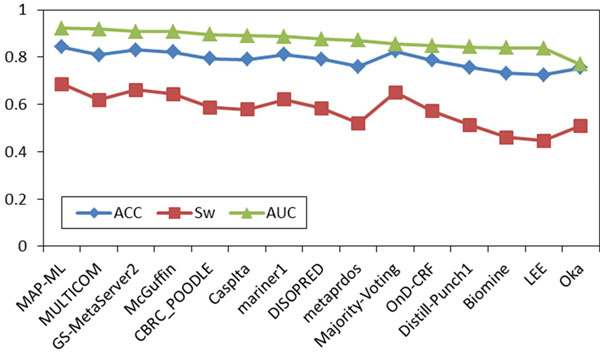
**CASP8 comparison on labeled data.** Evaluation scores are shown for the MAP-ML algorithm, majority voting method, and the 13 component predictors at disorder/order labeled CASP8 protein sequences and the corresponding experimentally determined disorder/order labels. ACC, S_w_, and AUC scores are sorted in descending order of the AUC score.

**Figure 5:**
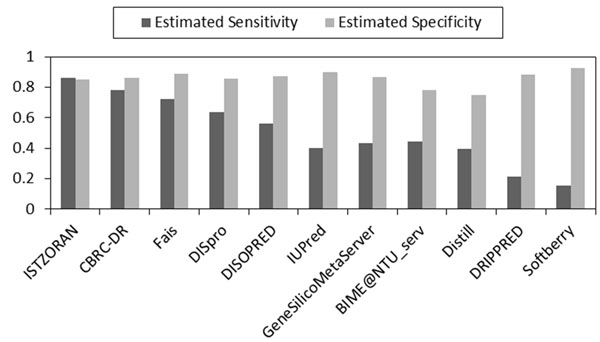
**CASP7 accuracy estimates without using labeled data.** Estimated sensitivity and specificity of 11 disorder predictors is obtained by the MAP-ML algorithm at CASP7 protein sequences without using CASP7 experimentally determined disorder/order labels. The predictors are sorted in descending order of the average of the estimated sensitivity and specificity.

**Figure 6:**
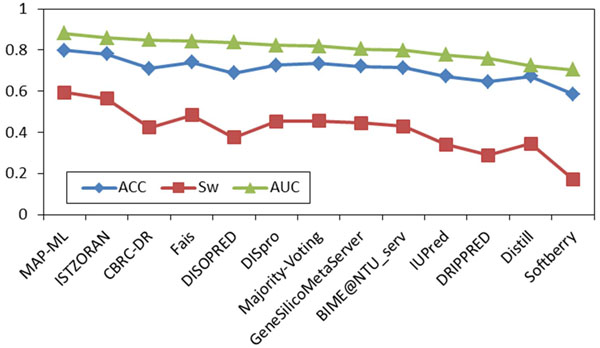
**CASP7 comparison on labeled data.** Evaluation scores are shown for the MAP-ML algorithm, majority voting method, and the 11 component predictors at disorder/order labeled CASP7 protein sequences and the corresponding experimentally determined disorder/order labels. ACC, S_w_, and AUC scores are sorted in descending order of the AUC score.

Using the same measures and procedures, we assessed the accuracy of 13 CASP8/11 CASP7 disorder predictors on CASP8 data [22]/CASP7 data [28] without using the corresponding experimentally determined disorder/order labels. Similar to CASP9, most of the predictors’ ranks obtained by the MAP-ML algorithm were quite consistent with their true accuracy on CASP8/CASP7 data. The scores of our MAP-ML meta predictor were better than the corresponding scores of component predictors in the CASP8/CASP7 contest and their majority voting integration. The details of the CASP8 experiment are summarized in Figure 3, Figure 4, and Table 2. The details of the CASP7 experiment are summarized in Figure 5, Figure 6, and Table 3.

**Table 2:** CASP8 evaluation scores on labeled data.

Predictor Name	Institution	ACC	S_w_	AUC
MAP-ML		0.843	0.686	0.922
GS-MetaServer2	IIMCB in Warsaw, Poland	0.831	0.662	0.908
Majority-Voting		0.826	0.651	0.856
McGuffin	University of Reading, UK	0.822	0.644	0.908
mariner1	George Mason University, USA	0.811	0.621	0.886
MULTICOM	University of Missouri, USA	0.809	0.619	0.918
CBRC POODLE	CBRC, Japan	0.794	0.588	0.895
DISOPRED	University College London, UK	0.792	0.583	0.876
CaspIta	University of Padova, Italy	0.790	0.579	0.891
OnD-CRF	Umea University, Sweden	0.786	0.572	0.848
metaprdos	University of Tokyo, Japan	0.760	0.520	0.871
Distill-Punch1	UCD Dublin, Ireland	0.756	0.513	0.843
Oka	IPR RAS, Russia	0.755	0.509	0.768
Biomine	University of Alberta, Canada	0.731	0.461	0.840
LEE	KIAS, Korea	0.724	0.447	0.837

**Table 3:** CASP7 evaluation scores on labeled data.

Predictor Name	Institution	ACC	S_w_	AUC
MAP-ML		0.798	0.595	0.881
ISTZORAN	Temple University, USA	0.781	0.564	0.860
Fais	University of Tokyo, Japan	0.740	0.484	0.844
Majority-Voting		0.734	0.455	0.819
DISpro	UC Irvine, USA	0.726	0.453	0.822
GeneSilicoMetaServer	IIMCB in Warsaw, Poland	0.720	0.446	0.804
BIME@NTU_serv	National Taiwan University	0.715	0.429	0.798
CBRC-DR	CBRC, Japan	0.710	0.423	0.850
DISOPRED	University College London, UK	0.689	0.375	0.837
Distill	UCD Dublin, Ireland	0.673	0.346	0.724
IUPred	Institute of Enzymology, Hungary	0.672	0.342	0.777
DRIPPRED	Imperial College London, UK	0.646	0.290	0.758
Softberry	RHUL, UK	0.586	0.173	0.704

### The relationship between the number of component predictors and the prediction performance

Although our MAP-ML meta predictor outperformed each component predictor at CASP9, CASP8, and CASP7, in general it may not be the case that integration of all available component predictors is the best choice as some predictors may negatively influence the combination results. To analyze effects of possible combination choices on the accuracy of the MAP-ML algorithm, we studied the relationship between the number of component predictors and the prediction performance of different combinations among CASP9, CASP8, and CASP7 predictors.

**Figure 7:**
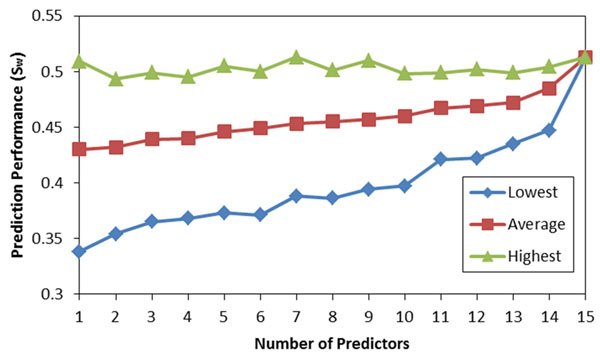
**The prediction performance of MAP-ML algorithm vs. the number of component predictors on CASP9 data.** The lowest, average, and highest performance for each group with the same number of individual predictors is shown.

**Figure 8:**
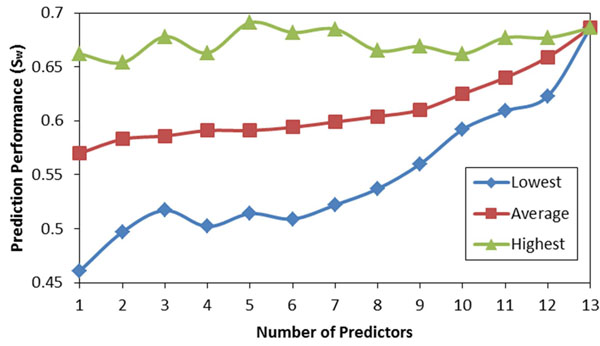
**The prediction performance of MAP-ML algorithm vs. the number of component predictors on CASP8 data.** The lowest, average, and highest performance for each group with the same number of individual predictors is shown.

**Figure 9:**
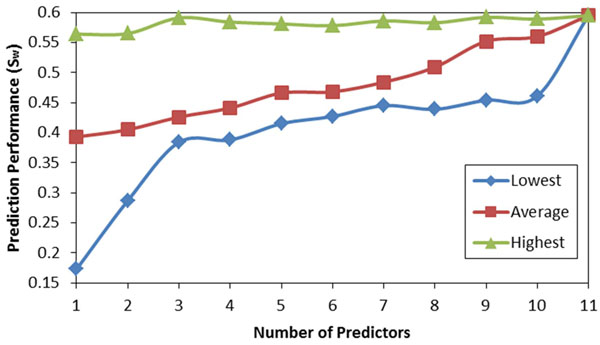
**The prediction performance of MAP-ML algorithm vs. the number of component predictors on CASP7 data.** The lowest, average, and highest performance for each group with the same number of individual predictors is shown.

For CASP9 data, any number out of 15 individual predictors can be combined by using our algorithm. By considering all subsets, we have constructed 32767 different meta predictors using the MAP-ML algorithm. The relationship between the number of component predictors and the prediction performance (S_w_) by the MAP-ML algorithm using CASP9 data is shown at Figure 7. Similarly, for CASP8/CASP7 data, we build all 8191/2047 meta predictors by considering all subsets of 13/11 component predictors and combining these using the MAP-ML algorithm. The relationship between the number of component predictors and the prediction performance (S_w_) by the MAP-ML algorithm using CASP8 and CASP7 data is shown at Figure 8 and Figure 9.

The results of our experiments (Figure 7, Figure 8, and Figure 9) provide evidence that the average and the lowest prediction performances improve as the number of component predictors increases. Also, the difference between the highest and the lowest performance decreases as the number of component predictors increases. However, the curves representing the highest prediction performances suggest that it is not the case that employing more component predictors will result in improved highest prediction performance. For example, a combination of five CASP8 predictors (MULTICOM, GS-MetaServer2, McGuffin, mariner1, and DISOPRED) had the highest overall prediction performance (S_w_=0.691).

## Conclusions

In this study, we proposed an iterative MAP-ML algorithm to predict protein disorder. The algorithm alternately provides the MAP estimation of disorder prediction and the ML estimation of the quality of multiple component disorder predictors. We evaluated the performance of the MAP-ML algorithm versus the performance of other predictors using CASP datasets. The results showed that our meta predictor not only outperformed other predictors but also appropriately ranked other predictors without knowing the true labels.

The proposed algorithm assumed that the accuracy of each predictor did not depend on the given protein sequences and that the predictors make their errors independently. Therefore, in our experiments we used the component predictors developed by groups at different institutions. We emphasize that in practice the independence assumption might not be always true, which is the limitation of the proposed algorithm. To relax the independence assumption and to make even more accurate disorder predictions by the probabilistic meta model, our research in progress includes additional parameters such as disorder flavor and difficulty of a prediction task.

## List of abbreviations used

CASP: Critical Assessment of Techniques for Protein Structure Prediction; DisProt: Database of Protein Disorder; PDB: Protein Data Bank; ROC: receiver operating characteristic.

## Competing interests

The authors declare that they have no competing interests.

## Authors' contributions

PZ designed the algorithms, implemented programs, carried out the analysis, and drafted the manuscript. ZO inspired the overall work, provided advice, and revised the final manuscript. All authors read and approved the final manuscript.
